# The S100 family is a prognostic biomarker and correlated with immune cell infiltration in pan-cancer

**DOI:** 10.1007/s12672-024-00945-x

**Published:** 2024-04-29

**Authors:** Xiaojie Liang, Xiaoshan Huang, Zihong Cai, Yeling Deng, Dan Liu, Jiayi Hu, Zhihao Jin, Xinyu Zhou, Hongsheng Zhou, Liang Wang

**Affiliations:** 1 Department of Hematology, Beijing Tongren Hospital, Capital Medical University, Beijing, 100730 China; 2 Department of Hematology, Nanfang Hospital, Southern Medical University, Guangzhou, 510515 China; 3 The First Clinical Medical College, Guangdong Medical University, Zhanjiang, 524000 China; 4 Department of Radiology, Shunde Hospital of Southern Medical University (The First People’s Hospital of Shunde), Southern Medical University, Foshan, China

**Keywords:** S100 protein family, Pan-cancer, Tumor microenvironment, Immune-excluded phenotype, Multi-omics analyses

## Abstract

**Background:**

The S100 protein family is a group of small molecular EF-hand calcium-binding proteins that play critical roles in various biological processes, including promotion of growth, metastasis and immune evasion of tumor. However, the potential roles of S100 protein family expression in tumor microenvironment (TME) cell infiltration in pan-cancer remain elusive.

**Methods:**

Herein, we conducted a comprehensive assessment of the expression patterns of the S100 protein family in pan-cancer, meticulously examining their correlation with characteristics of TME cell infiltration. The S100 score was constructed to quantify S100 family expression patterns of individual tumors.

**Results:**

The S100 family was a potent risk factor in many cancers. Clustering analysis based on the transcriptome patterns of S100 protein family identified two cancer clusters with distinct immunophenotypes and clinical characteristics. Cluster A, with lower S100 expression, exhibited lower immune infiltration, whereas, Cluster B, with higher S100 expression, featured higher immune infiltration. Interestingly, Cluster B had a poorer prognosis, likely due to an immune-excluded phenotype resulting from stromal activation. The analysis revealed robust enrichment of the TGFb and EMT pathways in the cohort exhibiting high S100 score, alongside a positive correlation between the S100 score and Treg levels, suggesting the manifestation of an immune-excluded phenotype in this group. Moreover, S100 families were associated with the prognosis of 22 different cancers and a noteworthy association was observed between high S100 score and an unfavorable response to anti-PD-1/L1 immunotherapy. Consistent findings across two independent immunotherapy cohorts substantiated the advantageous therapeutic outcomes and clinical benefits in patients displaying lower S100score.

**Conclusion:**

Our analysis demonstrated the role of S100 family in formation of TME diversity and complexity, enabling deeper cognition of TME infiltration characterization and the development of personalized immunotherapy strategies targeting S100 family for unique tumor types.

**Supplementary Information:**

The online version contains supplementary material available at 10.1007/s12672-024-00945-x.

## Introduction

In the context of tumor progression, conventional understanding has primarily focused on genetic and epigenetic alterations within tumor cells as the primary drivers. However, recent research has increasingly highlighted the vital role of the tumor microenvironment (TME) in supporting the growth and survival of tumor cells. Cancer cells do not operate independently; instead, they closely interact with both the extracellular matrix (ECM) and stromal cells within TME [1]. The TME is composed of various cellular components, including infiltrating inflammatory cells, carcinoma-associated fibroblasts (CAF), hematopoietic and endothelial progenitor cells, particularly in the early stages of tumor development [2]. In addition to immune cell infiltration, the TME also accommodates immunosuppressive cell populations, including myeloid-derived suppressor cells, regulatory T cells, and type 2-polarized macrophages, which hinder the anticancer immune response [3, 4]. Despite these challenges, immunotherapy approaches such as adoptive cell transfer and immune checkpoint inhibitors have significantly advanced cancer treatment and tumor immunology. Understanding the multifaceted and complex nature of the TME offers valuable insights into tumor progression, immune evasion, and the potential modulation of immunotherapeutic response. A thorough understanding of the intricate heterogeneity and complexity of the TME can facilitate the identification of diverse tumor immune phenotypes, thereby improving the prognostic value and predictive capacity of immunotherapeutic responsiveness. This comprehensive analysis also shows promise in identifying novel biomarkers that accurately determine which patients are likely to respond favorably to immunotherapy [5, 6].

The S-100 protein family is a low-molecular-weight protein family found in vertebrates. It is characterized by two calcium-binding sites with a spiral ("EF-hand") conformation. Members of the S100 family are divided into three major subgroups, each with different roles and functions: those that play only an intracellular regulatory role, those with intracellular and extracellular regulation, and those with a major role in extracellular functions. Under certain pathologic circumstances, a specific S100 protein may be expressed in a cell type that does not typically exhibit its expression [7]. The S100 family proteins have a wide range of functions in regulating cellular processes such as inflammation, apoptosis, energy metabolism, Ca2 + homeostasis, proliferation, differentiation, migration and/or invasion [8]. The expression of various members of the S100 protein family is commonly dysregulated in human cancers. Each type of cancer presents a distinct profile or signature of S100 proteins, which may vary depending on the stage or subtype of the cancer. For example, in non-small-cell lung cancer, the expression of S100A11 is increased, while in small-cell lung cancer, it is decreased. In the case of oral squamous-cell carcinoma, S100A7 is found to be expressed in the pre-invasive and well-differentiated stages, as well as in early tumors. However, its expression is not observed in non-invasive, poorly differentiated, and advanced tumors [9, 10]. In addition, the role of the S100 family in tumor progression and metastasis by modulating the tumor microenvironment (TME) has gained attention [11]. One example is the interaction between S100A7 and its receptor RAGE, which plays a crucial role in modulating the TME in breast cancer. This interaction is involved in the recruitment of matrix metalloproteinase 9 (MMP9)-positive tumor-associated macrophages (TAMs) [12]. Additionally, tumor cells can release various factors, including transforming growth factor-beta (TGF-β) and vascular endothelial growth factor (VEGF), which are capable of triggering the expression of S100A8/9 in stromal cells found in premetastatic lungs [13]. The comprehensive understanding of the role of S100 family proteins in TME and immunotherapy response is still lacking, although experimental studies have identified their significance for certain types of tumors.

This study analyzed multi-level data from 33 types of cancer, including point mutation, mRNA expression, protein expression, immune characteristics, and patient survival rate, to evaluate the expression and function of S100 family genes. The results suggest that the S100 protein family may increase the risk of cancer, and that an immune-excluded phenotype could be linked to poor prognosis in patients with high S100 expression patterns. Furthermore, a novel methodology for quantifying S100 family expression levels, called the S100 score, was developed. This score has the potential to serve as a prognostic biomarker and predictive factor for the effectiveness of immune-checkpoint inhibitors.

## Materials and methods

**Fig. 1:**
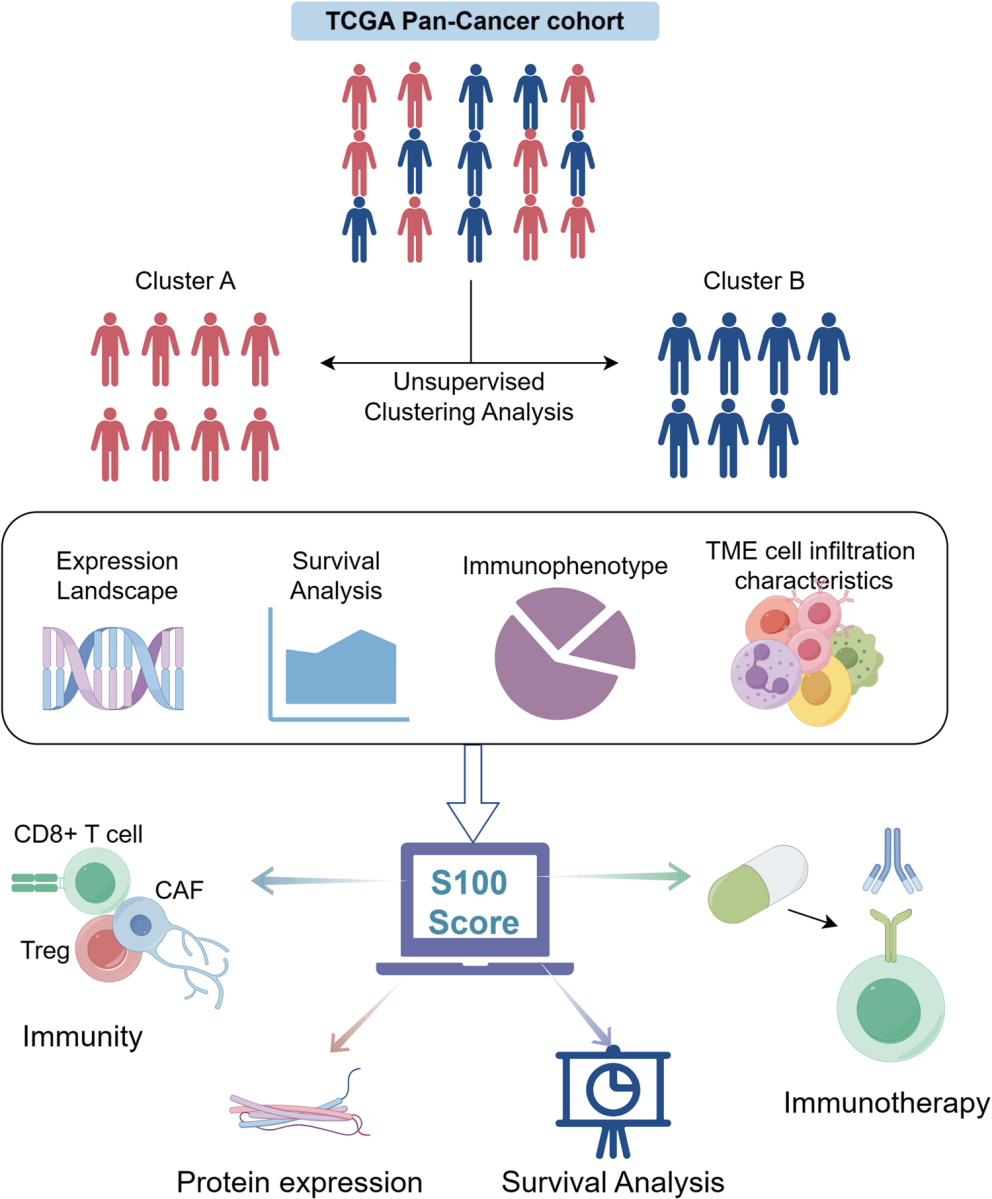
Schematic diagram of the study design

Figure 1 shows the workflow of this study.

### Dataset source and preprocessing

We systematically searched for publicly available datasets at all regulatory levels, ranging from genetic variation to transcriptomic profiles, for 33 cancer types, including adrenocortical carcinoma (ACC), bladder urothelial carcinoma (BLCA), breast invasive carcinoma (BRCA), cervical squamous cell carcinoma (CESC), cholangiocarcinoma (CHOL), colon adenocarcinoma (COAD), diffuse large B-cell lymphoma (DLBC), esophageal carcinoma (ESCA), glioblastoma (GBM), lower-grade glioma (LGG), head and neck squamous cell carcinoma (HNSC), kidney chromophobe (KICH), kidney renal clear cell carcinoma (KIRC), kidney renal papillary cell carcinoma (KIRP), acute myeloid leukemia (LAML), liver hepatocellular carcinoma (LIHC), lung adenocarcinoma (LUAD), lung squamous cell carcinoma (LUSC), mesothelioma (MESO), ovarian serous cystadenocarcinoma (OV), pancreatic adenocarcinoma (PAAD), pheochromocytoma and paraganglioma (PCPG), prostate adenocarcinoma (PRAD), rectum adenocarcinoma (READ), sarcoma (SARC), skin cutaneous melanoma (SKCM), stomach adenocarcinoma (STAD), testicular germ cell tumors (TGCT), thyroid carcinoma (THCA), thymoma (THYM), uterine corpus endometrial carcinoma (UCEC), uterine carcinosarcoma (UCS), and uveal melanoma (UVM).

The copy number variation (CNV) data was obtained from The Cancer Genome Atlas (TCGA) database, and the TCGA pan-cancer somatic mutation data (MC3 MAF v0.2.8 file) was downloaded from Genomic Data Commons (GDC, https://portal.gdc.cancer.gov/). RNA sequencing data (Counts value) of gene expression, as well as related clinical data, were downloaded using the R package TCGAbiolinks, which was specifically developed for integrative analysis with GDC data [14]. Pan-cancer RNA expression profiles (TPM value) in the TCGA database were obtained via the UCSC cancer browser (https://xenabrowser.net/datapages/). The Log2 transformed TPM was used for analysis. mRNA expression data from the Cancer Cell Line Encyclopedia (CCLE) were downloaded from https://portals.broadinstitute.org/ccle/.

Data on immunotyping, tumor type, tumor stage, treatment response, OS and OS time were retrieved from UCSC (https://pancanatlas.xenahubs.net/). Clinical information of pan-cancer samples has been integrated in Supplementary Table 1. Gene expression data and corresponding clinical data for Acute Lymphoblastic Leukemia (ALL) were downloaded from the Gene Expression Omnibus (GEO; https://www.ncbi.nlm.nih.gov/geo/, accession number GSE11877). Gene expression data and clinical data for ALL were also downloaded from the Therapeutically Applicable Research to Generate Effective Treatments (TARGET; https://ocg.cancer.gov/, phase II). Tumor-infiltrating immune cell abundance was calculated using CIBERSORT (file TCGA.Kallisto.fullIDs.cibersort.relative.tsv) and obtained from GDC [15].

### Overview of the S100 family

S100 family members are defined by a recent overview [10]. We conducted a comprehensive analysis of biological functions and effects of S100 family members, using a venn diagram to summarize the results. We calculated Spearman correlation coefficients between the expression of 21 S100 family genes in pan-cancer samples using the “corrplot” package. To obtain a global overview of correlations between S100s and cancer prognosis, we employed Cox regression to create survival landscapes for S100s across various cancer types.

### Genetic alterations analysis

To gain deeper insights into the phenomenon, we performed genetic alteration analyses. CNV data was analyzed using Genomic Identification of Significant Targets in Cancer (GISTIC) 2.0, which calculated a score based on the amplitude and frequency of CNAs at each probe position [16]. Somatic mutation information was analyzed using the “maftools” package to generate a mutation map for each family member [17].

### Unsupervised clustering analysis

Unsupervised clustering using K-means was applied to TCGA data (log2-TPM values) to identify S100 expression patterns and classify patients. The ConsensusClusterPlus R package was utilized and the procedure was repeated 1,000 times for classification stability [18]. The Wilcoxon test was used to compare RNA expression profiles (log2-TPM values) between the two clusters. Tumor sample distributions in each cluster were also determined. Prognostic analysis was conducted by generating Kaplan–Meier survival curves using the Survival and Survminer R packages. Clinicopathologic characteristics, including immunotyping, tumor type, tumor stage, and treatment response, were depicted and compared between the two clusters.

### mRNA expression analysis and the S100 score

To assess the dysregulation of S100 expression in different tumor types compared to normal tissue, we calculated log2 (fold changes) across 17 cancer types with at least five normal controls using the limma R package [19]. We also derived an S100 score by Z-normalizing the log2-TPM values of the 21 S100 genes in each cancer type, and computing the mean across these genes for each sample. The resulting S100 score provides a relative and comprehensive measure of S100 family members.

### Immunomodulators and immune cells analysis

To compare the immune profiles of the two clusters, we employed the ssGSEA (single-sample gene-set enrichment analysis) algorithm to evaluate the prevalence of TME-infiltrating cells in each sample. The gene sets used for identifying each TME-infiltrating immune cell type were obtained from a prior study [20], which included various subtypes of human immune cells such as activated B cells, activated CD4 T cells, macrophages, regulatory T cells, natural killer T cells, and monocytes, among others (see Supplementary Table 2). The enrichment scores derived from the ssGSEA analysis provided a quantitative measure of the relative abundance of each infiltrating cell type within the TME.

We identified a repertoire of immunomodulators such as antigen presentation factors, ligands, receptors, and other proteins based on a recent TCGA immune response working group publication [15]. To discern the relationship between these modulators' mRNA expression and the S100 score, we independently computed Spearman's correlation coefficients for each cluster.

We used the CIBERSORT gene expression deconvolution algorithm to estimate the relative proportions of 22 functional subsets of immune cells, for each cancer type [21]. The immune fractions were obtained from GDC [15]. The correlation between the immune fractions and the S100 score was then calculated across 33 cancer types.

### Functional and pathway enrichment analysis

To identify specific cellular functions and pathways enriched in the study, we conducted gene annotation enrichment analysis using the clusterProfiler R package [22]. Adopting the limma R package, we recognized differentially expressed genes (DEGs) between the two clusters based on an adjusted P value < 0.05 significance threshold. We identified upregulated Gene Ontology (GO) terms employing strict criteria of P < 0.1 and false discovery rate (FDR) of less than 0.1, and downregulated GO terms using the threshold of P < 0.01 and FDR < 0.01.

Gene set variation analysis (GSVA) was also performed using the GSVA R package. GSVA is usually applied to estimate the variation in pathway and biological process activity in the samples of an expression dataset in a non-parametric and unsupervised method [23]. The gene sets of “c2.cp.kegg.v2023.1.Hs.symbols” were obtained from MSigDB database for running GSVA analysis. To identify relevant pathways and biological process activity changes, we conducted differential analysis of pathways based on the S100 protein family pattern of action. We considered changes with an adjusted P value less than 0.05 and logFC more than 0.1 as statistically significant.

Gene Set Enrichment Analysis (GSEA) was conducted [24]. Spearman correlation coefficients were calculated between S100 score and mRNA expression of all protein coding genes for each cancer type. Genes were ranked by their correlation coefficient and pre-ranked gene lists were analyzed against hallmark sets using the clusterProfiler R package. Pathways demonstrating consistent significant correlations (FDR < 0.05) across at least 50% of all cancer types (at least 17 cancer types) were reported.

We then divided samples across all CCLE cell lines into two patient groups based on the S100 score and the log2 (fold change) were calculated by the limma R package. We also identified biological pathways using GSEA with all transcripts ranked by the log2 (fold change) between the two groups.

### Principal component analysis

We conducted principal component analysis (PCA) for two clusters using 21 S100 family protein and stroma-activated gene set separately. The members in pathway “KEGG_ECM_RECEPTOR_INTERACTION” in the gene sets of “c2.cp.kegg.v2023.1.Hs.symbols” were defined as the stroma-activated gene set.

### Immunohistochemistry staining

We obtained immunohistochemical staining images of S100A2 in cancer and normal tissue from HPA (https://www.proteinatlas.org/). Antibody HPA062451, CAB047340, CAB002600 were used to stain S100A2. The sample details, as well as the general pathological annotations and results, were accessed from HPA.

### Immunofluorescence staining of cancer cells

We accessed immunofluorescence staining of the subcellular distribution of S100A2 within the nucleus, microtubules and endoplasmic reticulum (ER) of A-431 epidermoid carcinoma cells, U-251MG glioblastoma cells and U2OS osteosarcoma cells.

### Survival analysis

Overall survival Kaplan–Meier curves were generated using the Survival and Survminer R packages for each cancer (including ALL). We employed a logarithmic rank test to assess the statistical significance of survival disparities between two clusters, as well as between low and high expression groups for each type of cancer. The Survminer R package's 'surv_cutpoint' was used to identify the optimal cut-point for the S100 score and separate patients into the low and high groups based on this score. Cox regression was utilized with the Survival R package to examine the connection between 18 family member expression and survival, as well as to examine the relationship between S100 scores and survival, and the optimal cut-point was identified using the Survminer R package.

### Collection and statistical analysis of immune-checkpoint blockade genomic and clinical information

Genomic and transcriptomic data sets from patients with metastatic urothelial cancer[25] treated with the anti–PD-L1 agent atezolizumab, as well as patients with metastatic melanoma[26] treated with anti–PD-1 pembrolizumab, were obtained and analyzed to assess the predictive value of the S100 score. For IMvigor210 cohort, the complete expression data and detailed clinical annotations could be obtained from http://research-pub.Gene.com/imvigor210corebiologies, under the Creative Commons 3.0 License. The count value was transformed into the TPM value. Additionally, for GSE78220 cohort, the FPKM data of gene expression profiles was also converted to the more comparable TPM value.

To generate overall survival Kaplan–Meier curves, the Survival and Survminer R packages were utilized. The 'surv_cutpoint' function from Survminer determined the optimal cut-point for the S100 score, facilitating the division of patients into low and high groups based on this score. Subsequently, treatment responses between these two groups were compared.

## Results

### Landscape of S100 family members in pan-cancer

**Fig. 2:**
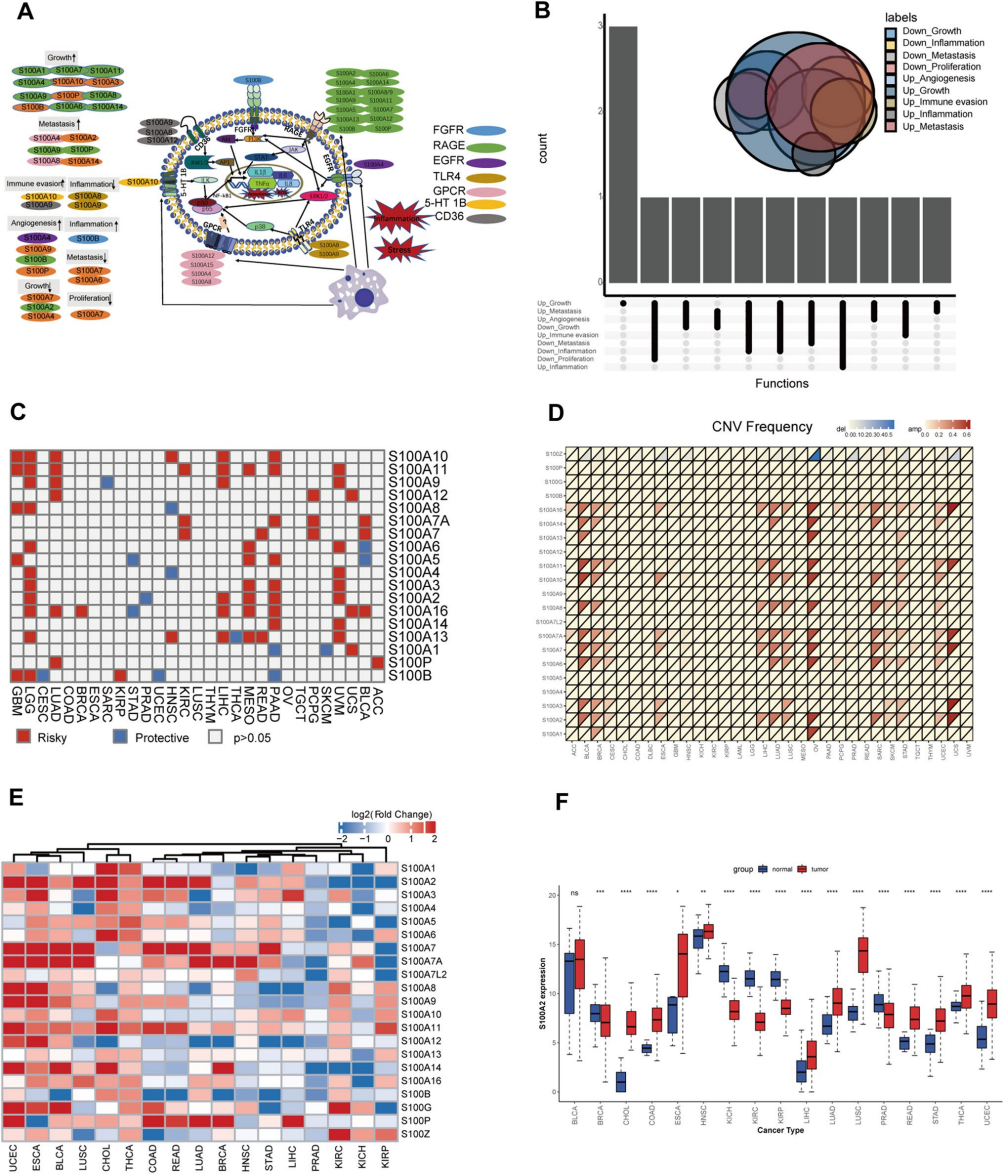
Pan-cancer genetic and expression alterations of S100 family members. **A** Diagram of the S100 protein family. **B** The proportion of S100 family members playing different roles in tumor progression. **C** Summary of the correlation between expression of S100 family members and patient survival, with red representing a higher expression of S100 family members associated with worse survival, and blue representing an association with better survival. **D** The CNV alteration frequency of S100 family members across 32 cancer types. The upper part of each grid shows the amplification frequency, and the bottom part shows the deletion frequency. **E** The gene expression alterations of S100 family members in 17 cancer types. The heat map shows the fold changes. Red represents upregulated genes, and blue represents downregulated genes. **F** Box plot showing the expression distribution of S100A2 across tumor and normal samples in 17 cancer types. *, P < 0.05; * *, P < 0.01; * * *, P < 0.001; * * * *, P < 0.0001, ns = no significance (Wilcoxon test)

The S100 protein family encompasses 21 members, participating in many biological processes, including autocrine and paracrine communication, orchestrate biological functions, as well as tumor development and progression in vivo (Fig. 2A). Focusing on the tumor development and progression, the reviewed 21 genes in S100 family from the pertinent literature, were involved in promotion and inhibition of tumor growth, facilitation and impediment of metastasis, regulation of inflammatory responses, angiogenesis, and immune evasion (Fig. 2B).

Then we wondered whether S100 protein family members would affect overall survival of 33 types of cancer. Overall, all members of S100 protein family were associated with survival at least one cancer. Moreover, most components were found to be risk factors rather than protective factors, such as S100A10, S100A11 (Fig. 2C). Interestingly, the landscape of CNV is marginally variable, while almost all S100s amplification could be observed in certain cancers, and S100Z was showed deletion in several cancers (Fig. 2D). Specific gene mutations for each member were shown in Supplementary Fig. 1. After that, the expression perturbation was explored across 17 cancers types compared with at least 5 normal controls (Fig. 2E). Most members were upregulated in certain cancer types, like UCEC, ESCA, and BLCA. Also, low expression levels were observed for almost all genes in certain cancer types such as LIHC, PRAD, and KICH. Notably, expression of S100A2 was seen to be significantly different across different cancer types, with increased expression in most cancers, but decreased in a few cancer types (BRCA, KICH, and KIRC) (Fig. 2F). Detailed expression of other family members was provided in Supplementary Fig. 2. Additionally, we explored the expression correlation between S100 family genes in pan-cancer (Supplementary Fig. 3). Most of the S100 family genes were positively correlated with each other, such as S100A4, S100A5, S100A6, S100A8 and S100A9; however, there were several genes that were negatively correlated with other family members, e.g., S100A1 was negatively correlated with S100P; whereas, S100G and S100A7L2 were weakly correlated with other family members. This demonstrated considerable genetic diversity and expression modulation of the S100 protein family in various cancer types, underscoring their pivotal involvement in disparate cancer scenarios.

### Expression landscape and clinical characters of two clusters generated by unsupervised consensus clustering

**Fig. 3:**
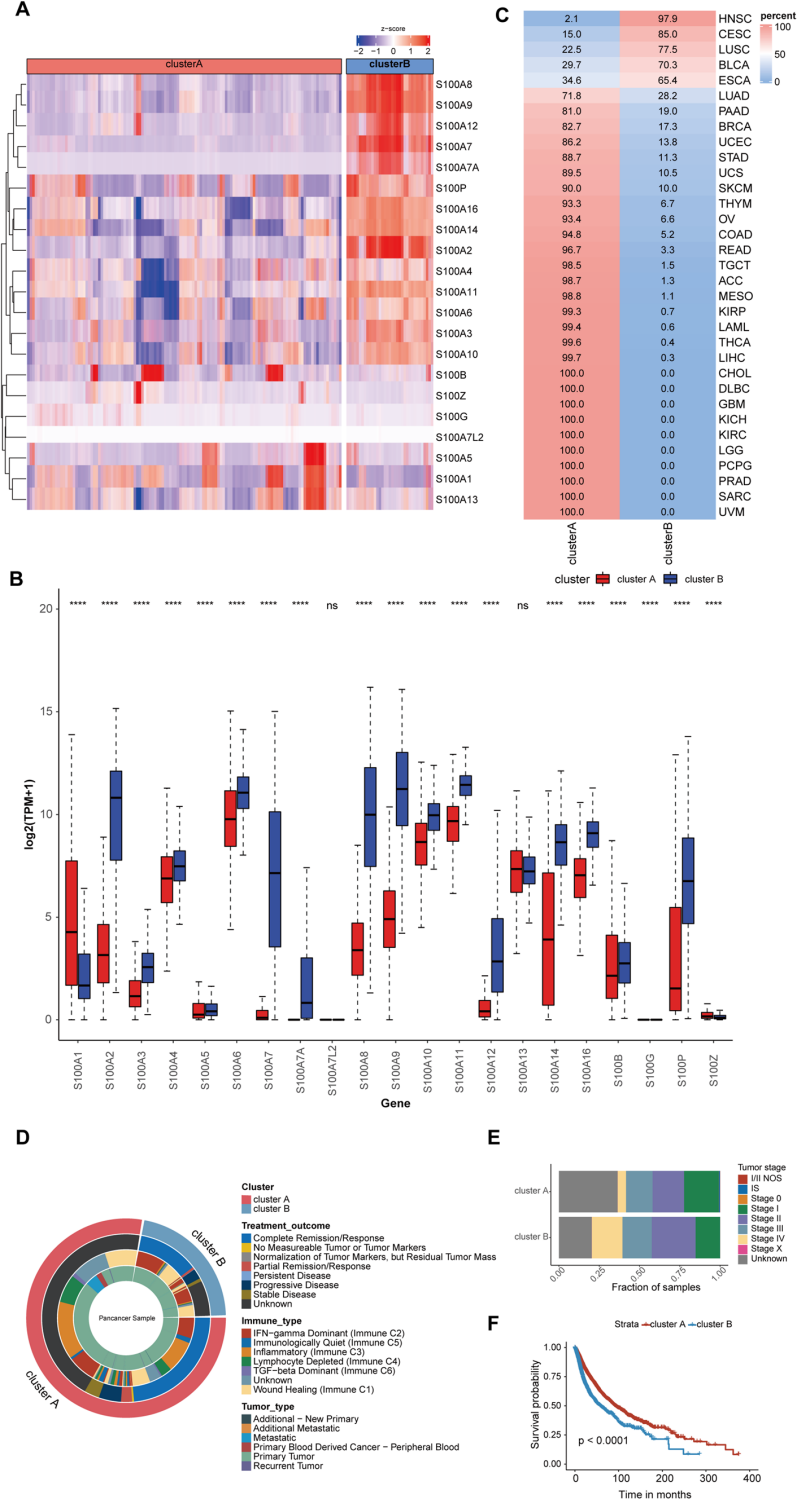
Expression landscape and clinical characters of two clusters generated by unsupervised consensus clustering. **A** Unsupervised consensus clustering of S100 family members gene expression proclaims two distinct clusters tagged by a different color in the top box. Each row represents a gene, and each column is a patient. Red indicates high expression, and blue indicates low expression. The expression data were normalized by z-score normalization for each row. **B** Boxplots showing expression of 21 S100 family members in two clusters. *, P < 0.05; * *, P < 0.01; * * *, P < 0.001; * * * *, P < 0.0001, ns = no significance (Wilcoxon test). **C** Sample distribution in the two clusters. Each row is a cancer type, and each column represents a cluster. The number in each box shows the percentage of samples sorted in the relevant cluster. **D** Overlay of clusters (outer ring) with clinical characters (inner rings) including treatment outcome, immune type and tumor type. **E** The proportion of samples with different tumor stage compared cluster A and cluster B. **F** Overall survival of patients between the two clusters in a Kaplan–Meier survival curve analysis. Statistical significance was assessed by log-rank test

To obtain a comprehensive expression landscape of S100 family members, unsupervised consensus clustering was performed and two distinct clusters of all tumor samples across 33 cancer types were identified (Fig. 3A). Obviously, almost all genes were highly expressed in cluster B (Fig. 3B). Interestingly, certain cancer types such as HNSC, CESC, LUSC, BLCA, and ESCA were predominantly enriched in cluster B, whereas the other 28 cancer types were mostly present in cluster A, indicating cancer type-specific dysregulation of S100 family gene expression (Fig. 3C).

We further compared the immunotyping, tumor type, and treatment response of two clusters and found significant differences between them (Fig. 3D). Also, a higher proportion of early-stage tumors was observed in cluster A (Fig. 3E), associated with a better survival outcome (Fig. 3F). This outcome may be attributed to a higher proportion of Inflammatory (Immune C3) immunophenotypes in cluster A, which has been previously linked to a favorable prognosis. Conversely, cluster B exhibited a predominance of IFN − gamma Dominant immunophenotypes (Immune C2), which were associated with worse outcomes, despite with a substantial immune component. [15]

### TME cell infiltration characteristics in distinct S100 expression patterns

**Fig. 4:**
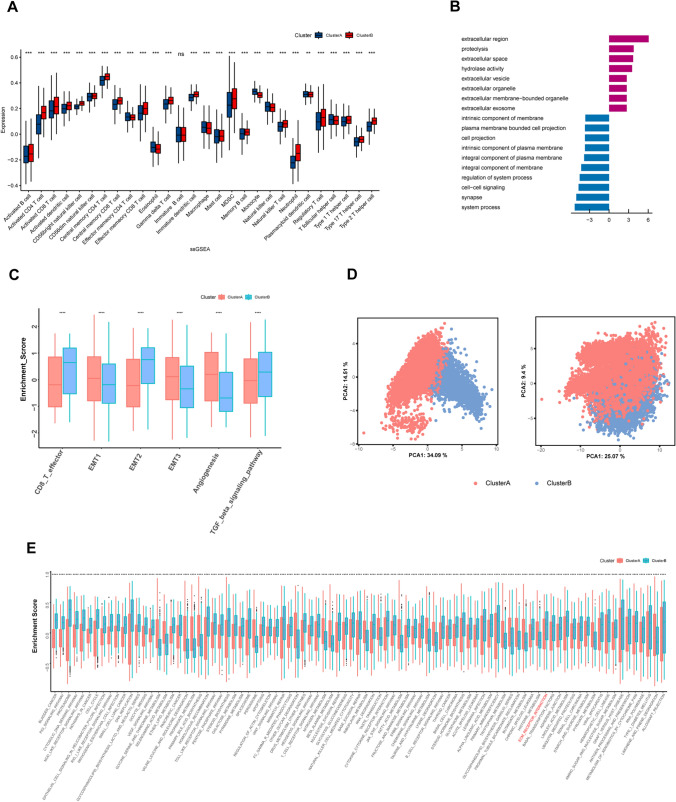
TME cell infiltration characteristics in distinct S100 expression patterns. **A** The abundance of each TME infiltrating cell in two S100 family expression patterns. The upper and lower ends of the boxes represented interquartile range of values. The lines in the boxes represented median value. The asterisks represented the statistical p value (*P < 0.05; **P < 0.01; ***P < 0.001, Wilcoxon test). **B** Bar plots showing the GO annotation of S100 family members. **C** Differences in stroma-activated pathways including EMT, TGF beta and angiogenesis pathways between two distinct S100 expression patterns. The asterisks represented the statistical p value (*P < 0.05; **P < 0.01; ***P < 0.001, Wilcoxon test). **D** Principal component analysis (PCA) of two distinct S100 expression patterns using 21 S100 family protein and stroma-activated gene set respectively, showing a remarkable difference on transcriptome and stroma activation state between different expression patterns. **E** GSVA enrichment analysis showing the activation states of biological pathways in distinct S100 expression patterns. The boxplot was used to visualize these biological processes. The upper and lower ends of the boxes represented interquartile range of values. The lines in the boxes represented median value. The asterisks represented the statistical p value (*P < 0.05; **P < 0.01; ***P < 0.001, Wilcoxon test)

Considering the conflict between the clinical survival and immune signature in cluster B, further analysis of TME cell infiltration was compared between two clusters. Notably, cluster B exhibited a significantly higher abundance of innate (mast cell, MDSC, plasmacytoid dendritic cell) and adaptive immune cells (activated B cell, activated CD4 T cell, activated CD8 T cell, Type 2 T helper cell, etc.) (Fig. 4A), although patients with this S100 expression pattern did not show a corresponding survival advantage (Fig. 3F). Previous studies have reported that tumors with an immune-excluded phenotype also exhibit a high number of immune cells but limiting migration of T cells through the tumor stroma, and affect the long-term survival [27]. Therefore, we proposed that stromal activation in cluster B may inhibit the antitumor effect of immune cells.

In order to further elaborate biological behaviors between these distinct modification patterns, we performed GO enrichment analysis for two clusters with different S100 expression levels. And the result indicated that cluster B was positively associated with extracellular pathways, such as extracellular vesicle and extracellular exosome (Fig. 4B). Extracellular vesicle activate the TGF-β signaling pathway [28], one of the critical hallmarks of T cell excluded tumors, contributing to changes in stroma [29]. These findings propose the S100 protein family as a key regulator of stromal activation.

Subsequent analyses confirmed that stroma activity was markedly enhanced in cluster B such as the activation of epithelial-mesenchymal transition (EMT) and transforming growth factor beta (TGFb), corroborating our hypothesis (Fig. 4C). We also performed GSVA enrichment analysis, which indicated that cluster B was enriched in stromal activation pathways, such as ECM receptor interaction (Fig. 4E). To investigate whether transcriptional profile of S100 protein family and stroma activating gene set can be distinguished between the two clusters, principal component analysis (PCA) was conducted for two clusters using 21 S100 family proteins and stroma-activated gene set separately. There was significant distinction existed on transcriptional level of S100 protein family and stroma activating gene set between two different S100 family expression patterns (Fig. 4D).

### Generation of the S100 score and its correlation with immunity

**Fig. 5:**
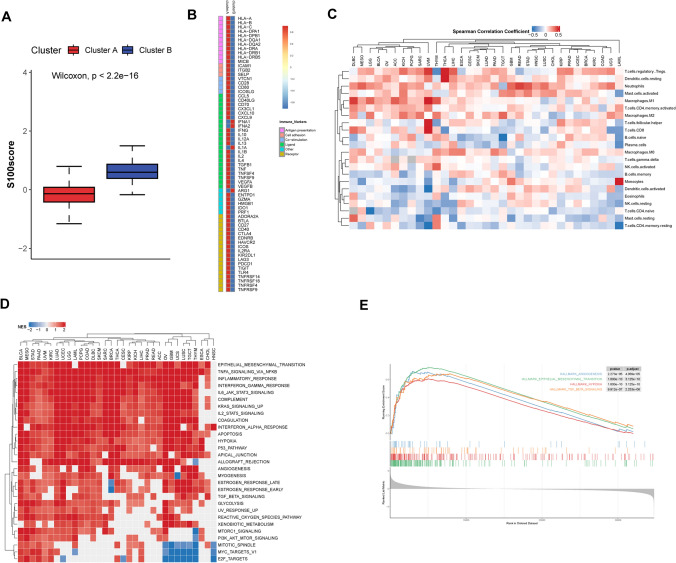
Generation of the S100 score and its correlation with immunity. **A** Box plot showing the S100 score in two clusters (Wilcoxon test). **B** Heat map representing spearman correlation coefficient between S100 score and immunomodulator expression for the two clusters. Red indicates positive correlation, and blue indicates negative correlation. **C** Heat map showing correlation between S100 score and levels of immune cells. Red indicates positive correlation, and blue indicates negative correlation. Unsupervised clustering used Euclidean distance metric with complete linkage. **D** Heat map showing normalized enrichment score of significant hallmarks sets in TCGA pan-cancer cohort. Each column is a cancer type, and each row is hallmark set. Red indicates positive normalized enrichment score, and blue indicates negative enrichment score. Significant hallmark sets are those with enrichment FDR < 0.05 in at least 17 cancer types (more than half of total number of cancer types). Unsupervised clustering used Euclidean distance metric with complete linkage. **E** Enrichment plots showing the angiogenesis (blue), EMT (green), hypoxia (red) and TGFb signaling (orange) gene sets in patient group with high S100 family expression in cancer cell lines from CCLE datasets

Taking into account the individual heterogeneity and complexity of S100 family modification, we devised an S100 score as a scoring system to quantify the S100 family expression pattern in individual patients with gastric cancer, using 21 S100 family protein. Cluster B, characterized with a high S100 expression pattern, exhibited a high S100 score (Fig. 5A).

To further validate the relationship between the mode of action of the S100 protein family and this unique tumor immune pattern, we conducted a series of analyses using the S100 score. We investigated the molecular association between the S100 score and immunomodulators separately in two clusters. In cluster B, correlation coefficients between S100 scores and a panel of key immunomodulators crucial for immunotherapy showed that the majority of immune markers, exhibited a negative correlation with S100 scores (Fig. 5B). These includes antigen presentation and co-stimulation markers, consistent with the attributes of the Immunophenotype Immune C2 [15]. This finding could elucidate the unexpected outcome observed in cluster B (Fig. 3F).

Then we investigated the association between the S100 score and immune cell abundance, including myeloid and lymphoid lineages in 33 different cancer types. Our analysis revealed significant positive correlations between S100 score and regulatory T cells, Dendritic cells resting, Neutrophils, Mast cells activated, Macrophages M0, Macrophages M1, T cells CD4 memory activated and Macrophages M2 across most cancer types. In contrast, we observed negative correlations between S100 score and Plasma cells, Monocytes, Dendritic cells activated, B cells memory, Eosinophils, NK cells resting, T cells CD4 memory resting, Mast cells resting, and T cells CD4 naïve (Fig. 5C).

In pathway analysis, the GSEA result showed that stroma activity was significantly enhanced in higher S100 expression level such as the activation of EMT and angiogenesis pathways (Fig. 5D). These correlations were further validated in CCLE data, an omic data of in vitro culture cancer cell lines. It proved that S100 expression was positively correlated with pathways such as EMT, angiogenesis, hypoxia and TGFb (Fig. 5E).

### The expression of S100 protein family across cancers

In our investigation at the transcriptome level, we uncovered distinct patterns of S100 family protein expression that correspond to varied tumor immune environments. To delve deeper into the matter, we are keen on elucidating the protein-level expression of S100 family protein across different cancer types. Given the widely disparate transcriptome-level expression of S100A2 between cancerous and normal tissue, we have opted for S100A2 as a representative candidate to illustrate the overall protein expression patterns of the S100 protein family.

**Fig. 6:**
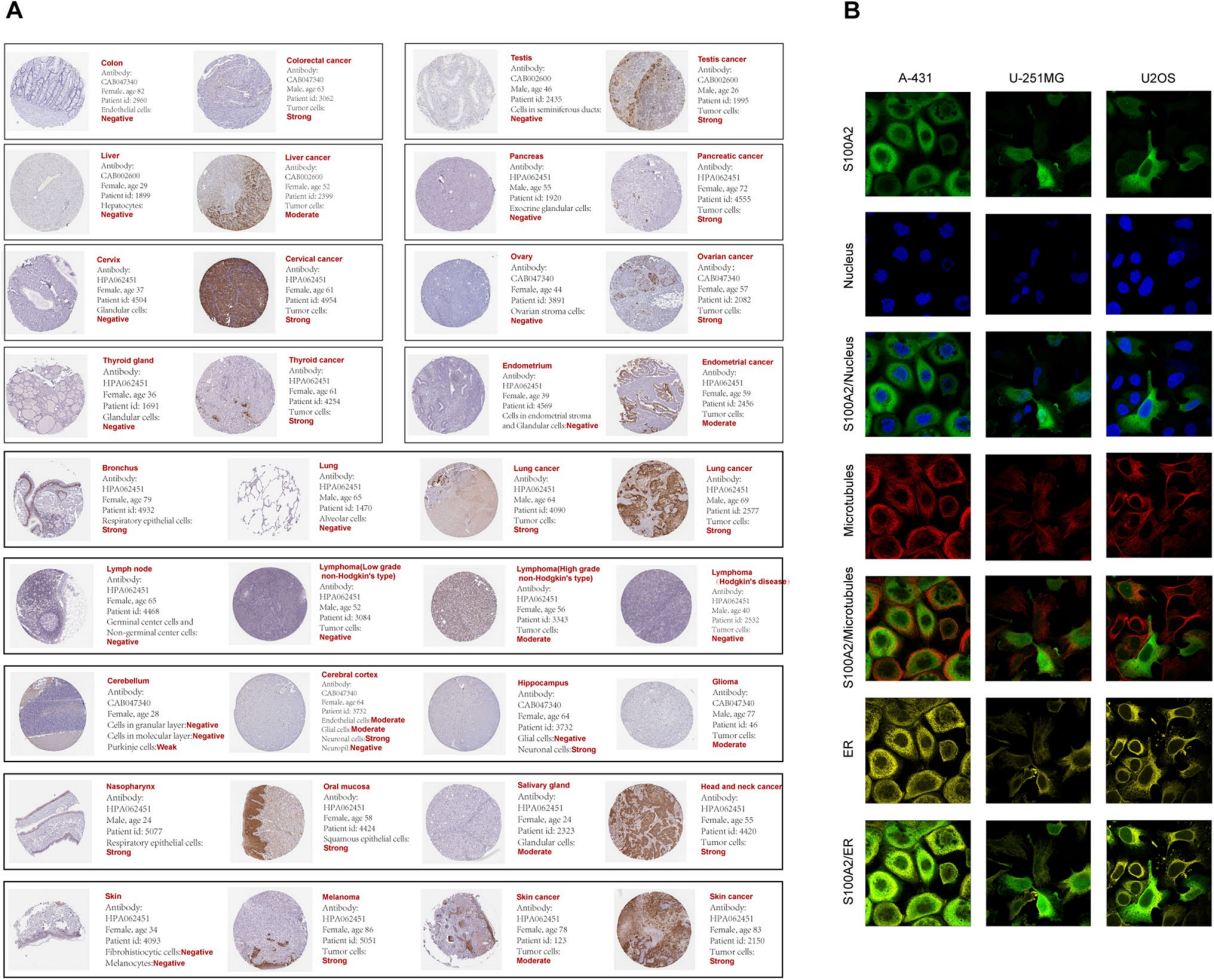
The expression of S100 protein family across cancers. **A** Protein staining images depicting the presence of S100A2 in cancerous and healthy tissues were captured from the Human Protein Atlas (HPA). The HPA provided comprehensive information regarding sample details and general pathological findings. **B** Immunofluorescence staining of the subcellular distribution of S100A2 within the nucleus, microtubules and endoplasmic reticulum (ER) of A-431 epidermoid carcinoma cells, U-251MG glioblastoma cells and U2OS osteosarcoma cells

We performed a comparative analysis of S100A2 staining patterns in cancer and non-cancerous tissues across 13 different tissue types. Visual representation of the data indicated that cancerous tissues exhibited higher intensity of S100A2 staining in 10 tissue types including colon, testis, liver, pancreas, cervix, ovary, thyroid gland, endometrium, head and neck cancer, as well as melanoma and skin cancer. Notably, both lung cancer and normal bronchus tissue displayed strong signals of S100A2 staining. Glioma exhibited a moderate S100A2 signal, while certain cell components of normal cerebral cortex and hippocampus tissues displayed strong signals. High-grade non-Hodgkin’s lymphoma demonstrated a moderate S100A2 signal, whereas lymph node, low-grade non-Hodgkin’s lymphoma, and Hodgkin’s lymphoma yielded negative S100A2 signals. Importantly, it is crucial to consider factors such as sample size, interindividual differences, and variations related to primary diseases, age, sex, and other pertinent parameters when interpreting these S100A2 staining data. Therefore, these findings should be regarded as reference information. (Fig. 6A).

We performed immunofluorescence staining to investigate the subcellular distribution of S100A2 in A-431 epidermoid carcinoma cells, U2OS osteosarcoma cells, and GBM cells. Our findings revealed predominant localization of S100A2 in the nucleoplasm and cytosol. Furthermore, S100A2 was observed in the nucleoli and plasma membrane. These results provide insights into the intracellular localization patterns of S100A2 in diverse cell types. (Fig. 6B).

### S100 family members predicts patient survival

**Fig. 7:**
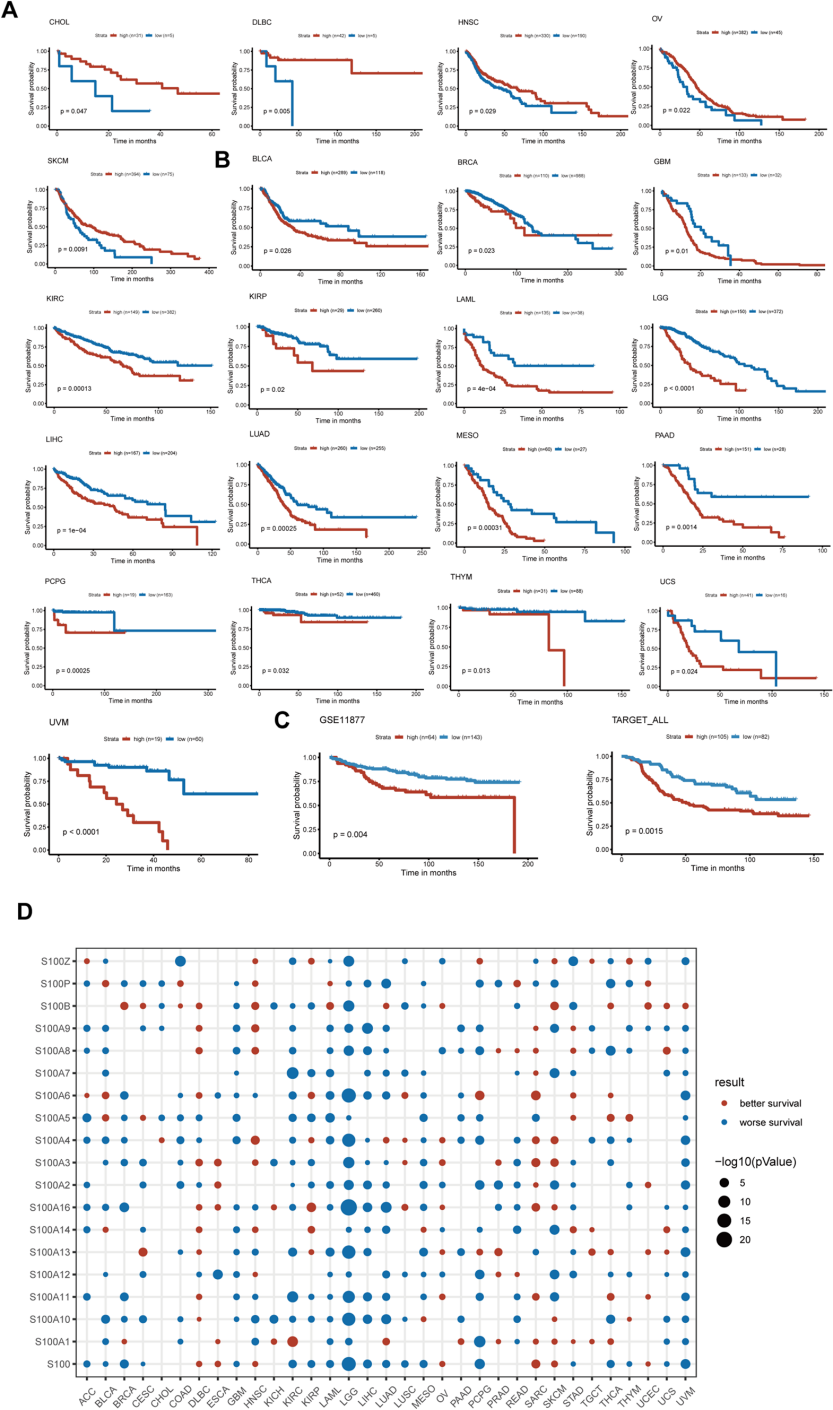
S100 family members predicts patient survival. **A**, **B** Impact of S100 expression on patient survival. High S100 score is associated with both better (**A**) and worse (**B**) overall survival. Overall survival of patients with high S100 score was compared with those with low S100 score in a Kaplan–Meier survival curve analysis. Statistical significance was assessed by log-rank test. **C** Influence of S100 expression on patient survival in ALL. **D** Summary of cox regression correlation of S100 with survival. Only significant dots with P value < 0.05 were shown. The size of dot is proportional to the − log10 (P value). Red indicates better survival, while blue indicates worse survival

The prevalence of genetic and expression modifications in S100 family members across different cancer types provides valuable insights into their clinical significance. Survival analysis of S100 family members in 21 cancer types (Fig. 7A and B) revealed that they served as a favorable prognostic factor in 5 cancer types (CHOL, DLBC, HNSC, OV, and SKCM) (Fig. 7A), while being associated with poor patient survival in 16 cancer types (BLCA, BRCA, GBM, KIRC, KIRP, LAML, LGG, LIHC, LUAD, MESO, PAAD, PCPG, THCA, THYM, UCS, and UVM) (Fig. 7B). Furthermore, our analysis of ALL data from GEO database indicated that S100 score was strongly associated with worse survival, suggesting that S100 family members could potentially be a risk factor in ALL (Fig. 7C).

The relationship between S100 expression level and patient survival was assessed using cox regression analysis for each of the 18 individual S100 family members and the S100 score, as shown in Fig. 7D. The S100 score was supported as an unfavorable prognostic biomarker in 19 tumors, including ACC, BLCA, BRCA, CESC, GBM, KIRC, KIRP, LAML, LGG, LIHC, LUAD, LUSC, MESO, PCPG, STAD, THCA, THYM and UVM. In particular, S100B was found to be linked to better survival in 13 cancers, while S100A10 was associated with worse survival in 19 cancers. In LGG, 17 S100 family members as well as the S100 score were found to predict worse survival. Overall, these findings highlight the predictive value of S100 family members in certain cancer contexts for patient survival.

### The S100 score predicts immunotherapeutic benefits

**Fig. 8:**
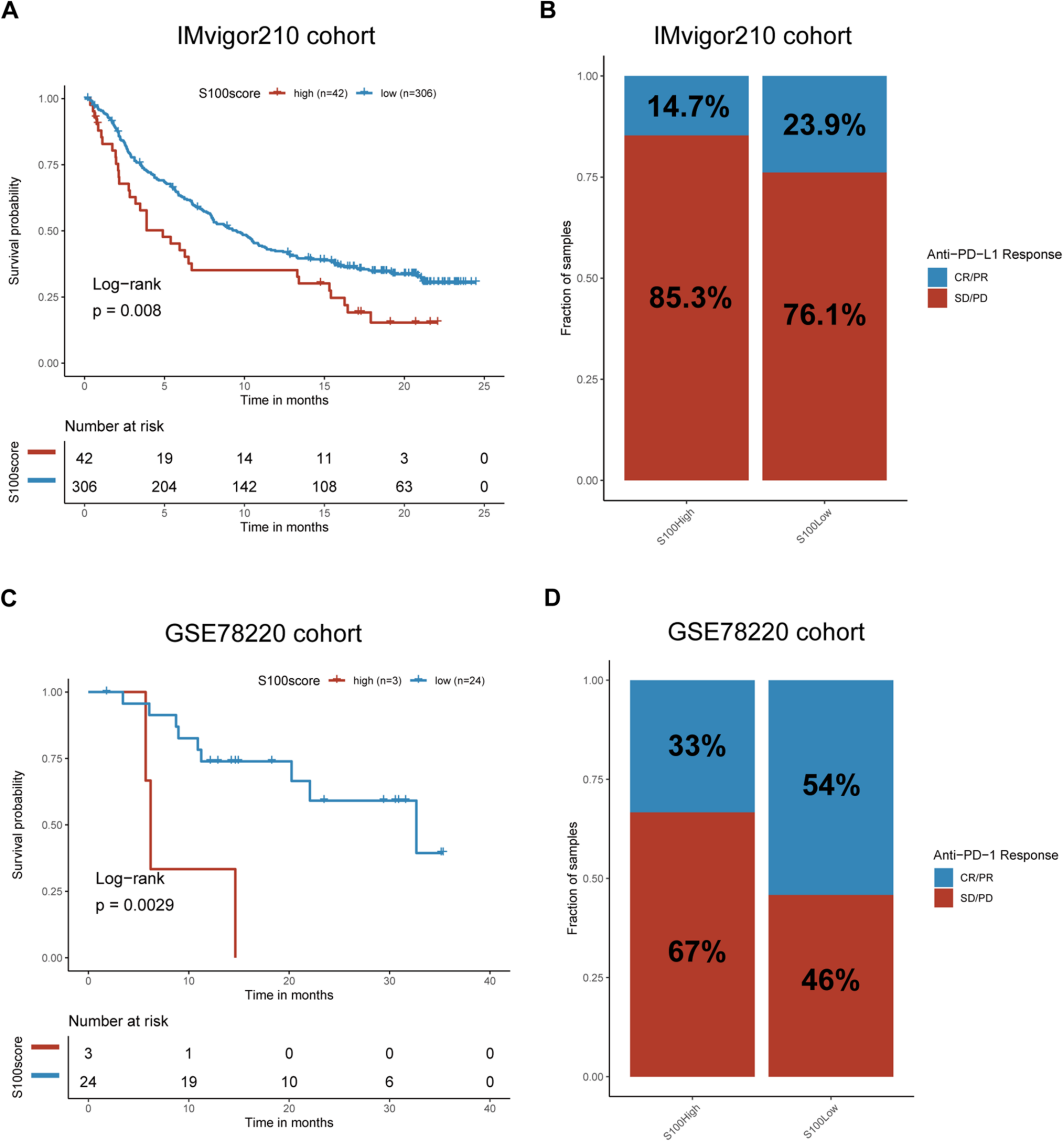
The S100 score predicts immunotherapeutic benefits. **A** Kaplan–Meier curves for patients with high (n = 42) and low (n = 306) TMEscores in the IMvigor210 cohort. Log-rank test shows an overall P = 0.008. **B** Rate of clinical response (complete response [CR]/partial response [PR] and stable disease [SD]/progressive disease [PD]) to anti–PD-L1 immunotherapy in high or low S100score groups in the IMvigor210 cohort. Patients with high S100scores: response (n = 5) and nonresponse (n = 29); patients with low S100scores: response (n = 63) and nonresponse (n = 201). **C** Kaplan–Meier curves for patients with high (n = 3) and low (n = 24) S100scores in the GSE78220 cohort. Log-rank test shows an overall P = 0.0029.** D** Rate of clinical response (CR/PR, SD/PD) to anti–PD-1 immunotherapy in high or low S100score groups in the GSE78220 cohort. Patients with high S100scores: response (n = 1) and nonresponse (n = 2). Patients with low S100scores: response (n = 13) and nonresponse (n = 11)

Therapeutic blockade of immunologic checkpoints through PD-L1 and PD-1 inhibitory monoclonal antibodies has emerged as a highly effective and synergistic anticancer strategy, offering unprecedented survival benefits [30]. To investigate the prognostic significance of S100 score in immune checkpoint therapy, we stratified patients from the IMvigor210 and GSE78220 cohorts into high and low S100 score groups. Patients with high S100 scores had worse survival and were less likely to benefit from immune-checkpoint therapy than those with lower S100 scores in both the IMvigor210 cohort (Fig. 8A, B) and GSE78220 cohort (Fig. 8C, D), which aligns with the immune-excluded phenotype observed in the cluster exhibiting high S100 family expression. In summary, our data suggest that the S100 score is in connection with response to different immunotherapy approaches.

## Discussion

The S100 protein family was well established to involve in cellular processes, including proliferation, apoptosis, differentiation, Ca2 + homeostasis and inflammation. Several S100 family members were verified to link to cancer, inflammatory disorders, and also neurological diseases [9]. Despite diversity and importance of S100 proteins was widely recognized, their complex roles are not yet fully understood. Therefore, we reviewed article published previously and found that almost all members involved in an array of process of cancer growth. Through the comprehensive analysis of TCGA multi-omic profiling data across 33 cancer types, we provided a global perspective on the regulation of S100 proteins in cancer.

Our genetic analysis indicated that S100s showed differential expression (upregulation or downregulation) between tumors and matched normal controls, furthermore, they have variable expression level across different cancer types. For example, almost all members of the S100 family were highly expressed in ESCA, consistent with previous studies demonstrating high expression of S100A7, S100A8, and S100A9 in esophageal squamous cell carcinoma (ESCC) [31, 32], which predicted cancer cell migration and worse overall survival. Silencing of the S100 proteins in co-cultured ESCC cells was found to suppress migration and invasion via Akt and p38 MAPK signaling pathways, as reported in previous studies [32]. Another interesting finding was that S100s were risk factor than protective factor. Multiple prior studies have corroborated a negative correlation between the S100 protein family and the prognostic outlook of pancreatic cancer, early-stage non-small cell lung cancer, and colorectal cancer [33–35]. Hence, our investigation is centered on elucidating the underlying factors linking S100 to a deleterious clinical outcome.

Herein, two patient clusters were distinguished based on expression patterns of S100 family, with nearly all S100 proteins displaying elevated expression in cluster B. This cluster exhibited a poorer prognosis (P < 0.001) and less favorable clinical features. Interestingly, cluster B possessed a greater proportion of C2 immunophenotype, suggesting that the poor prognosis observed in cluster B may be attributed to immune evasion mechanisms via immune editing [15].

To further elucidate this phenomenon, we focused on distinct TME cell infiltration characterization occurred in the two clusters. Cluster B, characterized by the activation of innate and adaptive immune cells, as well as stroma, exhibited an immune-excluded phenotype. Despite the abundance of immune cells in the TME, they were unable to penetrate the tumor parenchyma and were instead confined to the stroma surrounding tumor cell nests. The stroma might be limited to the tumor capsule or could penetrate the tumor itself, making it look as if that the immune cells are in reality inside the tumor [27, 36, 37]. In line with the above definitions, we observed cluster B exhibited a significant stroma activation status, including the highly expressed EMT pathways and TGF-β pathways, which were regarded as T-cell suppressive. The stromal compartment within the TME comprises non-tumor cells, including diverse fibroblast lineages that secrete distinct extracellular matrix (ECM) proteins, thereby modulating T cell migration. TGFb induces fibroblast activation and ECM production, creating a formidable physical impediment that hinders T cell infiltration [29]. In addition, the TGF-β signaling pathways encompass critical functions in driving the generation of cancer-associated fibroblasts (CAFs) [38]. Subsequent activation of these pathways also induces a remodeling of the extracellular matrix (ECM), culminating in the generation of biochemical and mechanical stimuli that enhance cancer cell invasion [39–42]. Preclinical studies examining advanced cancer models have previously shown that the activation of TGFb- and EMT-related pathways, along with fibroblast proliferation, leads to inhibition of T cell-mediated tumor killing and reduced T-cell mobility within the tumor [25, 43]. Coinhibition of TGF-β and PD-L1 shifts tumours from an excluded to an inflamed state, providing evidence for a model in which TGF-β signalling limits anti-tumour immunity via regulation of T-cell activity within the TME [25]. Our finding indicated the involvement of the S100 protein family in cancer progression by activating the stromal microenvironment. For instance, S100A7 induced an increase secretion of numerous factors, such as VEGF, granulocyte– macrophage colony-stimulating factor and angiopoietin, which act as central mediators of matrix remodeling, angiogenesis and inflammation, which led to a more aggressive milieu [44]. Therefore, it is not surprising that cluster B, despite having activated immunity, had a poorer prognosis.

Given the intricate heterogeneity evident in the expression of the S100 protein family, a pressing need arose to quantitatively assess the individual tumor's distinctive patterns of S100 expression. For that, we devised a comprehensive scoring system, denoted as the S100 score, to evaluate the unique S100 family expression profile for each patient. Cluster B, characterized by high S100 family expression, displayed a notable inverse relationship between S100 scores and various immune markers encompassing antigen presentation and co-stimulatory markers. Our investigation revealed a significant positive association between S100 scores and regulatory T cells as well as resting dendritic cells, manifesting across a broad range of cancer types. Regulatory T cells (Tregs) play a pivotal role in supporting tumor growth through suppression of the effector immune response [45]. In a radiation-induced pulmonary fibrosis model, Tregs were observed to facilitate EMT through the activation of β-catenin signaling [46]. Similarly, in a pancreatic cancer model, the deletion of Tregs resulted in decreased expression of Col and Fn1 mRNA in CAFs, accompanied by increased infiltration of effector CD4 + and CD8 + T cells. This phenomenon was attributed to the loss of TGF-β1, produced by Tregs. A potential hypothesis suggests that Tregs are a source of TGFβ and promote the differentiation of fibroblasts into CAFs [47]. Collectively, these findings indicate that stromal Tregs contribute to the development of CAFs, EMT, and metastasis, resulting in a barrier to immune infiltration into tumors, which may lead to the poor prognosis of the patients with high S100 family expression.

Having exploring the characteristics of two groups of patients distinguished by different S100 protein family transcriptome patterns, our attention shifted towards scrutinizing the protein-level expression of this particular family. At the protein level, mirroring the transcriptome, S100A2 exhibits heightened expression in tumors compared to normal tissues, providing additional evidence for the S100 protein family's potential as a prognostic risk factor. Our investigation elucidated the subcellular localization of S100A2, primarily within the nucleoplasm and cytoplasm. The existing body of research that highlights S100A2's predominant nuclear localization lends credence to its proposed function as a tumor suppressor [48].

To gain further insights into the clinical implications of S100 proteins, we examined the correlation between S100 scores and 34 cancer types, 22 of which were found significant for prognosis. In comparison to the S100-low cohort, individuals in the S100-high cohort demonstrated markedly inferior rates of survival across 17 types of cancer (Fig. 6B, D, P < 0.05). Our analysis indicates the significant prognostic value of the S100 family in LGG, aligning with earlier observations demonstrating the unfavorable prognostic implications of S100A8, S100A9, and S100A11 in LGG [49, 50]. In addition, our findings reveal a poorer prognosis in individuals with high S100 scores amongst ALL patients, thus corroborating previous literature citing that the downregulation of S100A6 expression via amlexanox can effectively reduce resistance to TNFα and impairs tumor growth in KMT2A/ aff1-positive ALL [51]. However, the effects of S100 family proteins on the prognosis of ALL patients and their mechanisms need to be further explored.

Previous investigations have implicated the activated stromal TME in immune resistance towards immune-checkpoint blockade therapy, while also highlighting its potential impact on individualized cancer immunotherapy. Here, we present evidence elucidating the substantial role of S100 protein family expression patterns in delineating diverse stromal and immune TME landscapes. Our findings underscore the significance of S100 protein family expression in modulating the therapeutic effectiveness of immune checkpoint blockade. In two distinct patient cohorts with metastatic urothelial cancer[25] receiving anti-PD-L1 (atezolizumab) treatment and metastatic melanoma receiving anti-PD-1 (pembrolizumab) treatment, we have substantiated the predictive capacity of the S100 score for checkpoint blockade. Our findings reveal that patients with low S100 scores exhibited superior survival rates and more favorable responses to checkpoint blockade therapy compared to those with high S100 scores. These outcomes can be attributed to the immune-excluded phenotype that arises due to stromal activation induced by the S100 family. However, it may be practicable to control stromal composition. Notably, in the context of pancreatic cancer, agonistic anti-CD40 antibody has been reported to activate macrophages that enter the TME, remodel the stroma, and enhance the responsiveness of tumors to chemotherapy. [52] Therefore, targeting S100 family protein could be an effective strategy to remodel the stroma and facilitate the development of immunotherapy.

Our findings indicate that S100 acts as a prevalent cancer risk factor, with differing expression patterns linked to distinctive TME traits. A comprehensive appraisal of S100 expression patterns within individual tumors could enhance our comprehension of cell infiltration within TME and facilitate the development of more effective immunotherapy strategies. We derived a S100 score to be utilized for assessing patients’ responsiveness to immunotherapy and prognosis. We also shed light on regulatory variations of S100s occurring across different levels, including genetic alterations, gene expression, pathway correlations and protein expression. These discrepancies can lead to distinct treatment outcomes, and variable patient survival rates, which highlights the importance of individual based treatment according to the nature of cancer diversity. Future research could therefore focus on the validation of the S100 proteins as biomarkers in early disease detection and prognosis, and on the development of novel strategies based around anti-S100 therapies.

### Supplementary Information

Additional file1 (ZIP 1700 KB)

## Data Availability

The datasets analyzed in this study are available in the following open access repositories: GDC, https://portal.gdc.cancer.gov/ . UCSC, https://xenabrowser.net/datapages/. GEO https://www.ncbi.nlm.nih.gov/geo/ (GEO accession numbers: GSE11877). CCLE, https://portals.broadinstitute.org/ccle/. TARGET, https://ocg.cancer.gov/. Other datasets are available from the corresponding author upon reasonable request.
