# Supplementary figures and images for: The S100 family is a prognostic biomarker and correlated with immune cell infiltration in pan-cancer
Source: Discov Oncol. 2024 Apr 29;15:137. doi: 10.1007/s12672-024-00945-x (PMC11058162; doi:10.1007/s12672-024-00945-x)

# Supplementary Figure 1

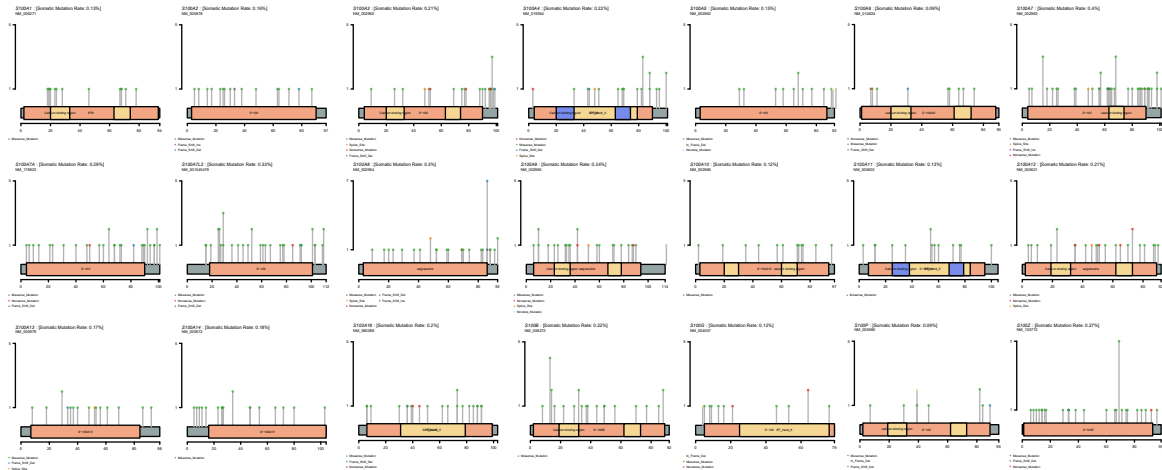

Supplement: Supplementary file 1 — Additional file1 (ZIP 1700 KB) [file 12672_2024_945_MOESM1_ESM.zip › Figure S1.pdf]

**Supplementary Figure 2**

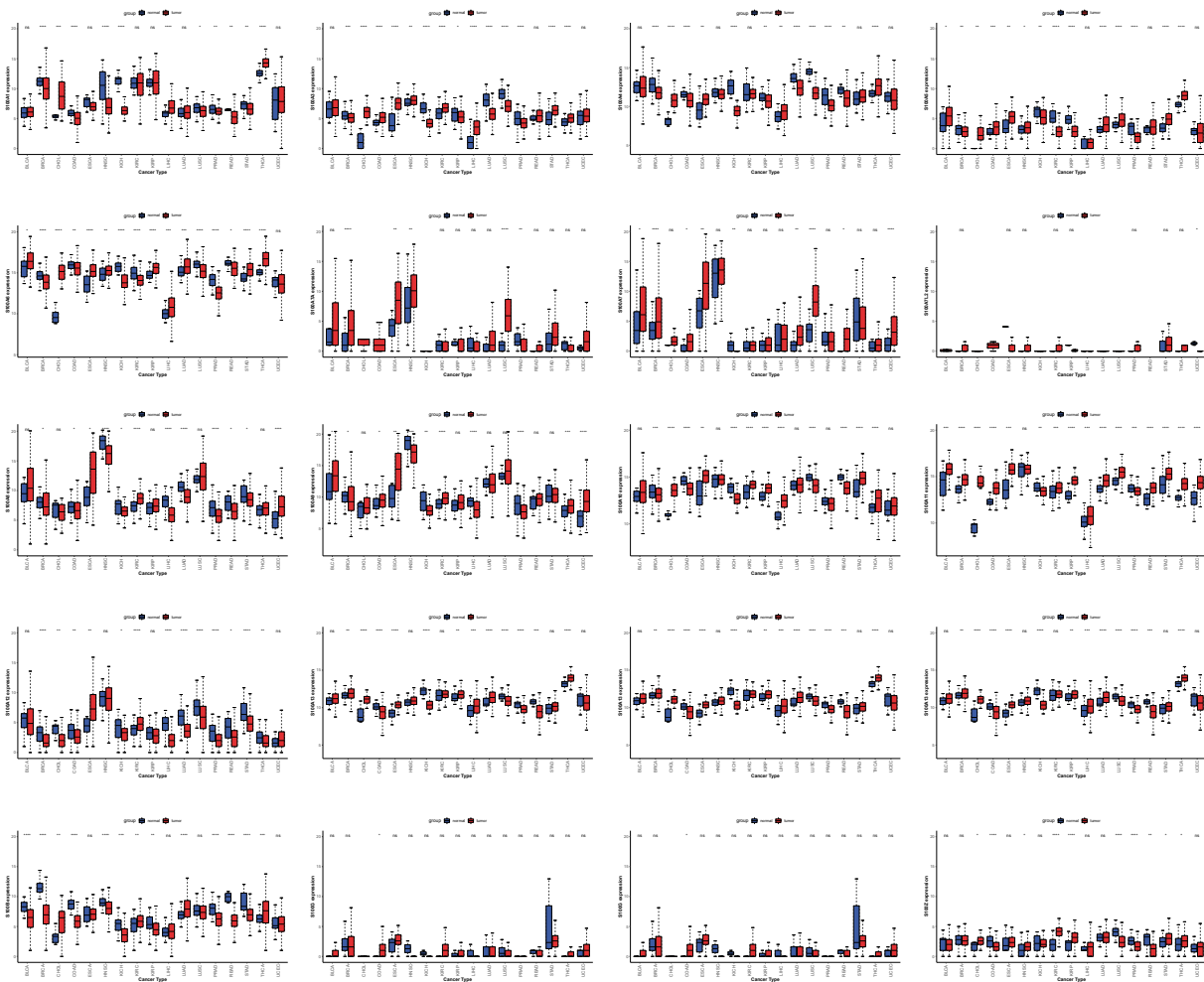

Supplement: Supplementary file 1 — Additional file1 (ZIP 1700 KB) [file 12672_2024_945_MOESM1_ESM.zip › Figure S2.pdf]

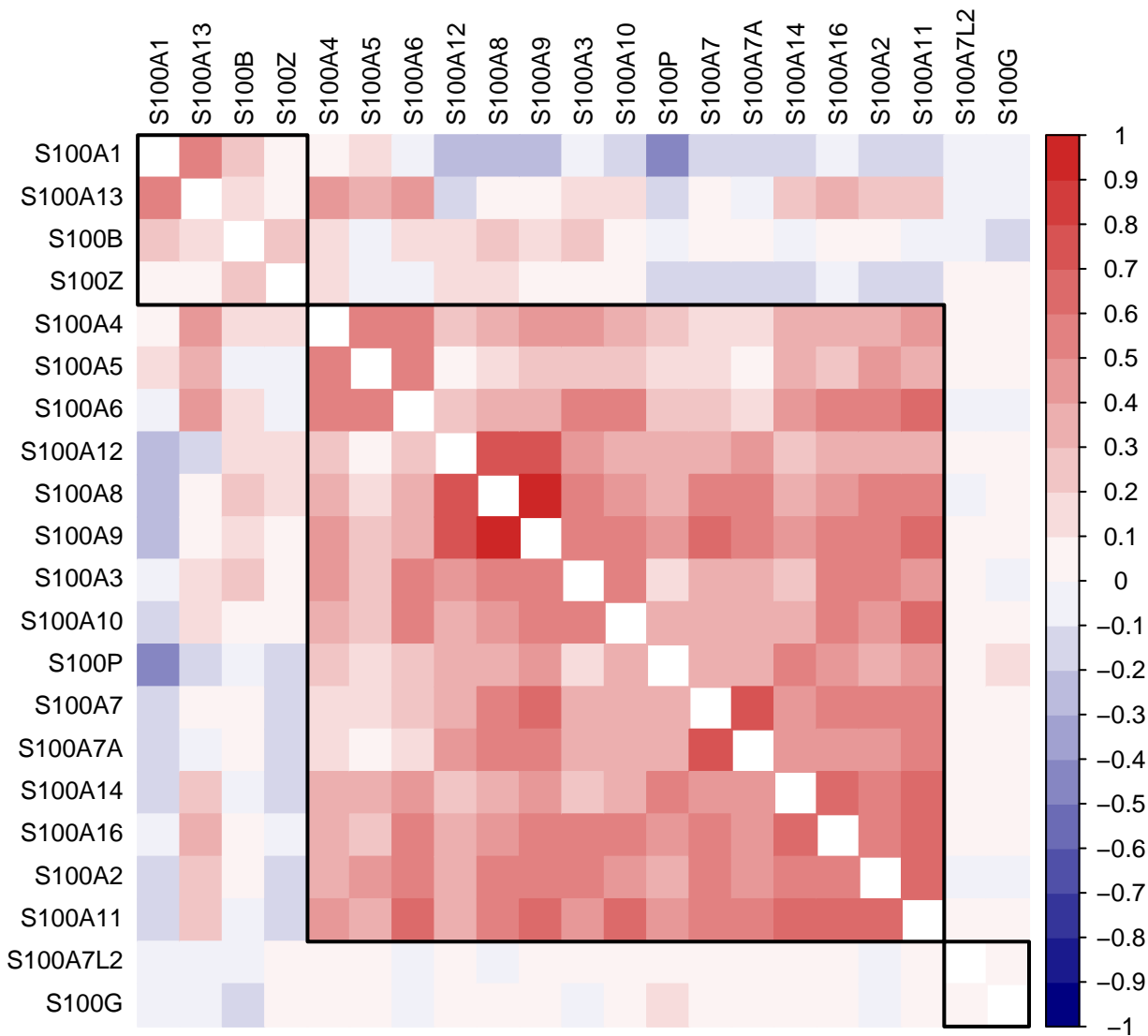

Supplement: Supplementary file 1 — Additional file1 (ZIP 1700 KB) [file 12672_2024_945_MOESM1_ESM.zip › Figure S3.pdf]
